# Antibody Responses against Enterovirus Proteases are Potential Markers for an Acute Infection

**DOI:** 10.3390/v12010078

**Published:** 2020-01-09

**Authors:** Niila V. V. Saarinen, Virginia M. Stone, Minna M. Hankaniemi, Magdalena A. Mazur, Tytti Vuorinen, Malin Flodström-Tullberg, Heikki Hyöty, Vesa P. Hytönen, Olli H. Laitinen

**Affiliations:** 1 Faculty of Medicine and Health Technology, Tampere University, FI-33014 Tampere, Finland; niila.saarinen@tuni.fi (N.V.V.S.); virginia.stone@ki.se (V.M.S.); minna.hankaniemi@tuni.fi (M.M.H.); malin.flodstrom-tullberg@ki.se (M.F.-T.); heikki.hyoty@tuni.fi (H.H.); vesa.hytonen@tuni.fi (V.P.H.); 2 Karolinska Institutet, The Center for Infectious Medicine, Department of Medicine Huddinge, Karolinska University Hospital, 14152 Stockholm, Sweden; magdalena.mazur@ki.se; 3 Turku University Hospital, Clinical Microbiology and University of Turku, Institute of Biomedicine, 20520 Turku, Finland; tyvuori@utu.fi

**Keywords:** enterovirus, acute infections, proteases

## Abstract

Background: Enteroviruses are a group of common non-enveloped RNA viruses that cause symptoms ranging from mild respiratory infections to paralysis. Due to the abundance of enterovirus infections it is hard to distinguish between on-going and previous infections using immunological assays unless the IgM fraction is studied. Methods: In this study we show using Indirect ELISA and capture IgM ELISA that an IgG antibody response against the nonstructural enteroviral proteins 2A and 3C can be used to distinguish between IgM positive (*n* = 22) and IgM negative (*n* = 20) human patients with 83% accuracy and a diagnostic odds ratio of 30. Using a mouse model, we establish that the antibody response to the proteases is short-lived compared to the antibody response to the structural proteins in. As such, the protease antibody response serves as a potential marker for an acute infection. Conclusions: Antibody responses against enterovirus proteases are shorter-lived than against structural proteins and can differentiate between IgM positive and negative patients, and therefore they are a potential marker for acute infections.

## 1. Introduction

Enteroviruses are non-enveloped positive stranded RNA-viruses (Group IV viruses) that cause a wide variety of diseases ranging from mild respiratory infections to severe conditions such myocarditis, meningitis, encephalitis, flaccid paralysis, and systemic “septic” infections in young infants [1]. In addition to acute infections, enteroviruses have been linked to the development of chronic diseases such as cardiomyopathies, asthma, and autoimmune diseases including type 1 diabetes where persistent viral infections may also be involved [2,3,4,5,6]. Enteroviruses (mainly EV-71 and CAV16) are also the main causative agents of hand-foot-and-mouth disease [7]. Enterovirus infections are frequent in all age groups causing significant morbidity particularly in young children and are a heavy health-economic burden on society [8].

Reliable diagnosis of enterovirus infections is a prerequisite for offering patients effective treatment and for the avoidance of unnecessary antibiotic prescriptions. There are no licensed antivirals available but some drugs including Pleconaril and immunoglobulins have been trialed in the treatment of severe infections [1]. The lack of, or wrong diagnosis of infections often results in the misdiagnosis of enterovirus infections as bacterial infections and subsequent treatment with antibiotics [9,10], which underscores the need for tests that can specifically detect acute enterovirus infections. Enterovirus infections are currently diagnosed mainly by the detection of viral RNA in clinical specimens using RT-PCR. This method is highly sensitive and specific but requires specialized laboratories and sophisticated equipment. Another limitation with applying RT-PCR-based diagnostics on blood samples is that viremia is short-lived, and viruses can only be detected for a few days [11]. Serological methods can overcome this problem since antibody responses last longer and said assays also require less sophisticated laboratory facilities. Enterovirus infections can be diagnosed by either detecting enterovirus specific IgM class antibodies in acute-phase samples or through demonstrating significant increases in IgG antibody titers in paired sera. Both of these assays have been used in clinical diagnostics, but their wider use has been curbed by limited sensitivity in their ability to detect antibodies induced by over one hundred enterovirus types and, in the case of IgG assays, the need for paired sera which is a challenge for use in the clinical diagnostics of infections. Thus, there is a clear need for better serological assays to improve the clinical diagnostics of enterovirus infections.

The enterovirus genome is expressed as a single polyprotein that is cleaved into structural (capsid) and non-structural proteins, such as the proteases. Non-structural proteins are not included in the virion and only appear when the RNA-genome is translated in the host cell. The non-structural proteins carry out multiple functions that aid with viral replication and immune response evasion strategies during infection [12]. The enterovirus proteases 2A and 3C are essential non-structural protein components in the life cycle of a virus as they are responsible for cleaving the virus polyprotein into its subunits.

Historically, serological assays have used structural capsid proteins or purified whole viruses as antigens, while antibody responses against non-structural proteins are less well characterized. However, antibody responses to non-structural proteins can offer advantages in clinical diagnostics. First, these proteins are more conserved among different enteroviruses than the structural proteins [13], providing an option to detect antibodies against several different enterovirus types. Second, since non-structural proteins are only produced during an active virus infection, antibodies against them could be used to distinguish vaccine-induced antibodies from those that have been induced by natural infections. This would be advantageous when analyzing the efficacy of inactivated enterovirus vaccines. In fact, antibodies against non-structural proteins, such as 2C and 3ABC have been routinely used for screening livestock to distinguish between foot and mouth disease (FMD) infected and FMD vaccinated animals [14,15]. Antibodies against non-structural enterovirus proteins have also been found in humans during acute EV-71 and CAV16 infections, especially against 3A, 3C and 3D [7,16]. The differing IgG antibody responses towards structural and non-structural proteins have been used to differentiate acute and chronic hepatitis C virus infections (another Group IV virus) [17], and varying antibody response profiles have also been connected to the severity of enterovirus 71 infections [7].

In the present work, our aim was to study the nature of IgG antibody responses against the non-structural enteroviral proteins 2A and 3C and to compare these responses to those raised to the enteroviral structural capsid proteins. We set out to study these responses in human patients with IgM-confirmed acute enterovirus infections and in control subjects, as well as in enterovirus-infected mice. The results suggest that the analysis of antibodies against viral proteases provides information that helps to diagnose acute enterovirus infections.

## 2. Materials and Methods

### 2.1. Animal Husbandry and Ethics Statement

C57BL/6J mice were housed in isolator cages under SPF conditions at the Karolinska University Hospital Huddinge, Stockholm, Sweden. Infections were performed according to local and national regulations, and the study was approved by the Stockholm South Animal Ethics Board (Södra Djurförsöksetiska nämnd; Number: S46-14; Approval date: August 29th 2014, 5-year approval, the animal work with subsequent analyses were performed within this period).

Animals were infected with Coxsackievirus B3 (CVB3) or mock-infected with PBS. Blood and tissue samples were collected at different times for histological analysis and ELISA (See Appendix A for details).

### 2.2. Human Samples Ethics Statement

Enterovirus IgM positive serum samples were originally tested in the virus diagnostic laboratory in Turku University Hospital for the diagnosis of patients’ acute enterovirus infections. The samples were anonymised and used in the development of antibody tests. Control serum samples included laboratory personnel and the participants of the Autoimmune defence and living environment-study (ADELE) [18] and negative control samples from 6-month old children who were participating in the Diabetes prediction and prevention-study (DIPP) [19]. All participants or their legal guardians gave informed consent.

### 2.3. Preparation of ELISA Antigens

The VP1 antigen (CVB3, strain Nancy, original sequence from GenBank: M33854.1) were prepared as described in [20]. The production of recombinant proteases 3C and 2A (CVB3, strain Nancy, original sequence from GenBank: M33854.1) was carried out as in [21]. CVB3 was grown in green monkey kidney cells (Vero) as described previously [6,22]. The adenovirus and parainfluenzavirus antigens were obtained from Heikki Hyöty’s laboratory. See Appendix A for details on antigen preparation.

### 2.4. Protease ELISA

Conditions for protease ELISA were optimized using pooled mouse sera and Nanogam^®^ before running individual mouse and human samples. Samples were run with a typical indirect ELISA protocol. See Appendix A for details on antigen preparation.

### 2.5. Capture IgM-ELISA for Human Samples

Enterovirus IgM antibodies were analyzed by a standard method used in clinical virus diagnostic laboratory in the Turku University Hospital, as described by [23]. See Appendix A for details on antigen preparation.

### 2.6. Data Processing

The ELISA results from mice were analyzed in individual animals: The average signal for each antigen and dilution were divided by the same mouse’s corresponding pre-infection response to obtain a fold change in signal intensity. Average signal + 3 times standard deviation from parallel serum free wells for each antigen was subtracted from the raw data. The fold changes were then compared to the mock-infected controls using one-tailed Mann–Whitney’s rank test at different time points utilizing GraphPad Prism v.5.02.

The human data processing was done differently as there were no paired samples in the acute versus control dataset: Absorbance data (with serum free control signals subtracted) from the assay was used to compare the two groups. Wilcoxon signed-ran test was used for comparing groups. Data processing for the human samples was done using R version 3.4.4 with the following packages: ggplot2, ggpubr, GGAlly, ggExtra, reshape, cowplot, and tidyverse. Final layouts for figures were done using Inkscape version 0.92.3.

## 3. Results

### 3.1. Enterovirus Proteases Are Expressed in Mice Shortly after CVB3 Infection

**Figure 1:**
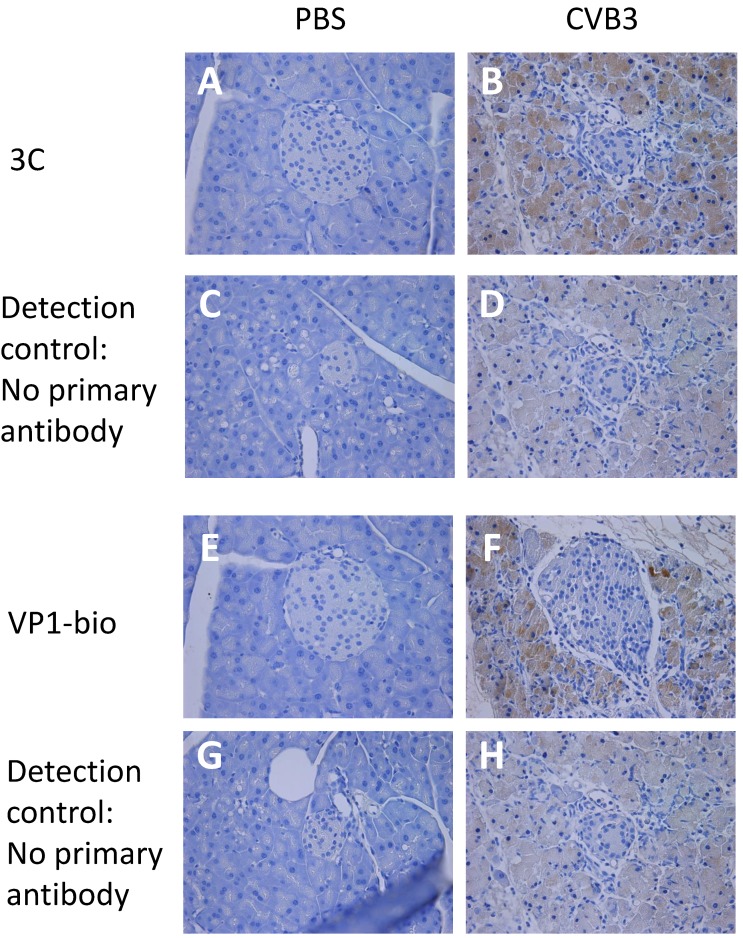
Viral 3C and VP1 proteins are expressed in murine pancreata following infection with Coxsackievirus B3 (CVB)3. C57BL/6J mice were mock-infected with PBS (**A**,**C**,**E**,**G**) or infected with CVB3 (10^2^ PFU/mouse) (**B**,**D**,**F**,**H**) for 3 days. Pancreas sections were stained with either an antibody detecting 3C (**A**,**B**) or a directly biotinylated antibody detecting VP1 (**E**,**F**). As control for the 3C staining, sections from mock-infected and CVB3 infected animals were stained with a biotinylated secondary antibody alone (**C**,**D**) prior to visualization of antibody binding by DAB as described in Materials and Methods section. For the VP1 controls, sections were incubated without secondary antibody prior to visualization of antibody binding (**G**,**H**). Representative sections are shown (*n* = 3–4 animals per condition). Positivity is indicated with the brown staining. Original magnification ×10.

In a previous study, we found that monoclonal antibodies can detect 2A and 3C in infected mouse tissue [21]. Here we found that these antibodies detected viral proteases in tissue samples taken early after infection, but not in samples collected at later time points (Figure 1). Pancreata from CVB3 infected mice collected 3 days post infection (dpi) showed positivity in the exocrine pancreas with both 3C and VP1 (1), while the staining was absent at time points after 7 dpi (Table S1).

### 3.2. Pooled Mouse and Human Sera Contain Antibodies against Enteroviral Proteases

**Figure 2:**
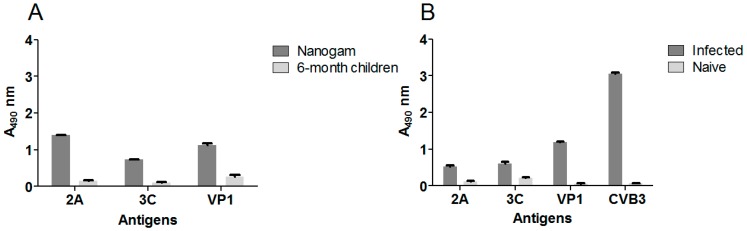
Antibody reactivity towards enterovirus structural and non-structural proteins in serum from 6-month old children, adults and CVB infected mice. (**A**) 2A, 3C, and CVB3 VP1 antibodies in sera pooled from 6 month-old humans (1:1000 dilution, white) and from Nanogam^®^ serum (1:10,000 dilution, gray bars) and (**B**) 2A, 3C, VP1, and CVB3 antibodies in pooled naïve (white bars) and CVB3 infected (21 dpi; grey bars) mouse sera (both 1:1000 dilution) as detected by ELISA. Each bar represents a pool of sera (*n* = 20 for humans, *n* = 18 for infected mice and *n* = 4 for naïve mice). Data is presented as means ± S.E.M.

In order to compare the profiles of the antibody responses against viral proteases to those against viral capsid proteins, VP1 and heat inactivated whole virions were used as antigens. Initially, we examined for the presence of 2A, 3C, VP1, or CVB3 antibodies in pooled human sera from (presumably naïve) 6-month old children, in a concentrated pool of adult human IgG (Nanogam^®^), and also in pooled sera from naïve or enterovirus infected mice (21 dpi) using ELISA (Figure 2). We saw that there was a strong antibody reactivity towards the proteases present in the Nanogam^®^ preparation, which was comparable to the antibody reactivity to structural proteins (Figure 2A). In comparison, pooled sera from six-month old children showed lower reactivity to the proteases (Figure 2A). Enterovirus (CVB3) infected mice also developed an antibody reactivity against viral proteases, which is lower in uninfected mice, yet, the antibody reactivity to the viral proteases was weaker than the response to the structural VP1 protein and the whole virus (Figure 2B). These results confirm that we can detect antibodies against the proteases in murine sera using ELISA which corresponded with protease expression in the pancreas by immunohistochemical analysis using protease-specific monoclonal antibodies.

### 3.3. Time Dependent Antibody Response against Enteroviral Proteases and Structural Proteins Differ in Mice

To better understand the kinetics of the antibody responses during an infection we decided to examine this in the controlled setting of a murine enterovirus infection model. Mice were infected with the enterovirus (CVB3) or mock infected with PBS. Blood samples were collected with 7-day intervals after the infection and analyzed for different enterovirus antibodies by ELISA.

**Figure 3:**
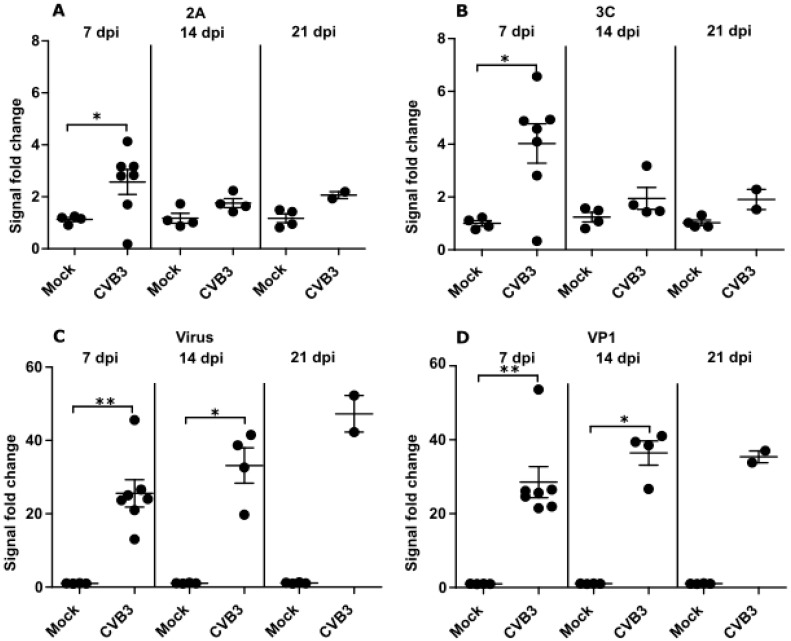
CVB-infection induces a short-lived antibody response towards CVB3 proteases in mice. Antibody responses towards the protease antigens (**A**) 2A and (**B**) 3C (at 1:1000 dilutions) and to the (**C**) structural proteins in whole virus and (**D**) the capsid protein VP1 (1: 64,000) in CVB3 infected mice measured using ELISA. The data is shown as the fold change compared to antibody responses from pre-infection serum samples. Each data point is an average of three parallel samples. Stat: Mann–Whitney test, means and standard errors of means shown as gray bars, * *p* < 0.05 and ** *p* < 0.001.

Differences between the antibody response towards the proteases and the structural proteins were detected (Figure 3). In enterovirus-infected mice, protease specific antibodies peaked at 7 dpi and declined to levels close to those seen prior to infection by 21 dpi (Figure 3A,B). The antibody responses against proteases only differed significantly (*p* < 0.05) from the control mice at 7 dpi. In comparison, the antibody response against the structural proteins and whole virus continued to increase until 14 dpi and remained significantly higher (*p* < 0.05) at this time point in CVB3 infected mice than in control mice (Figure 3C,D). Statistics for 21 dpi could not be calculated because some mice were euthanized prior to this time point leading to insufficient sample numbers. These results examining the duration of enterovirus antibody responses over time in mice suggest that upon infection, the protease antibody response is weaker and shorter in duration than the responses against the enterovirus structural proteins (See Table S2 for interassay variabilities for mice ELISA data).

### 3.4. Antibody Responses towards Viral Proteases during Acute Enterovirus Infection in Humans

**Figure 4:**
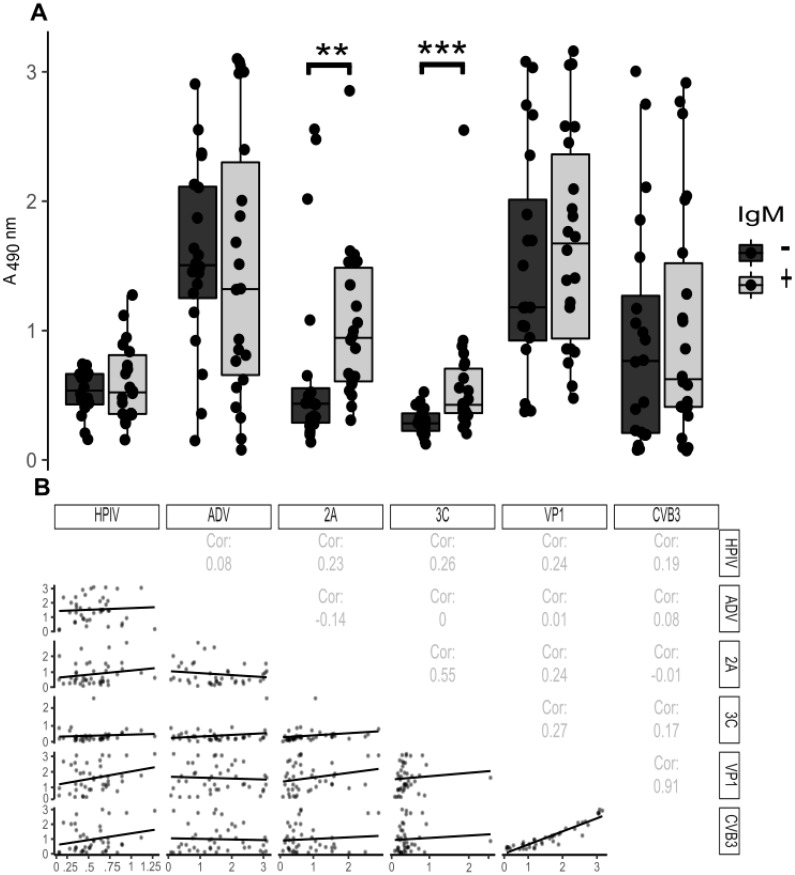
Antibody response towards viral proteins in acutely infected patients. Serum samples obtained from acutely infected humans with confirmed enteroviral infections (IgM positive, *n* = 22) and healthy controls (IgM negative, *n* = 20) were analyzed for immunoreactivity towards the indicated viruses or viral proteins. (**A**) Boxplot diagrams show the antibody responses towards different antigens analyzed using ELISA (serum diluted 1:1000) for acutely infected human patients (gray) and controls (black). ** *p* < 0.001 and *** *p* < 0.0001, two-tailed Wilcoxon signed-rank test analyzed in R. (**B**) The data set in A was used for creating a correlation matrix with pairwise comparisons. The top-right diagonal in panel (**B**) shows the Spearman correlation coefficients of antigen pairs and the bottom-left diagonal shows pairwise scatterplots with linear regression trend lines. Abbreviations: HPIV, human Parainfluenzavirus; ADV, Adenovirus; 2A, CVB3 2A protease; 3C, CVB3 3C protease; VP1, CVB3 VP1; CVB3, heat inactivated whole Coxsackie B3 virus.

Antibodies against the enteroviral proteases were analyzed in human serum samples which had been tested for IgM antibodies against enterovirus structural proteins. Samples taken from patients with an acute enterovirus-infection (IgM positive, *n* = 22) were used as well as randomly selected control subjects (IgM negative, *n* = 20). Sera from IgM positive, acutely infected patients had significantly higher reactivity for the 2A (*p* = 1.5 × 10^−3^) and 3C (*p* = 1.3 × 10^−4^) proteases when compared to healthy, IgM negative controls, while antibodies against structural proteins (whole virus particle and the VP1 protein) did not differ between the groups (Figure 4A). In addition, the parainfluenza and adenovirus antibody levels showed no difference between the two groups (Figure 4A).

Next, we studied whether there was any correlation between the antibody reactivity against different virus antigens. The responses to the 2A and 3C proteins had a moderate correlation (Spearman correlation (Cor) = 0.55) being higher than for any of the other antigen pairs, apart from whole virus and VP1, which expectedly had a strong correlation (Cor = 0.91) (Figure 3). Most importantly, only the responses against 2A and 3C show potential for distinguishing between the IgM positive and negative patient groups (Figure 4A) (See Table S3 for interassay variabilities for human ELISA data).

### 3.5. Sensitivity and Specificity of Detection of IgG against Viral Proteases in the Diagnosis of IgM-Positive Acute Enterovirus Infections

**Figure 5:**
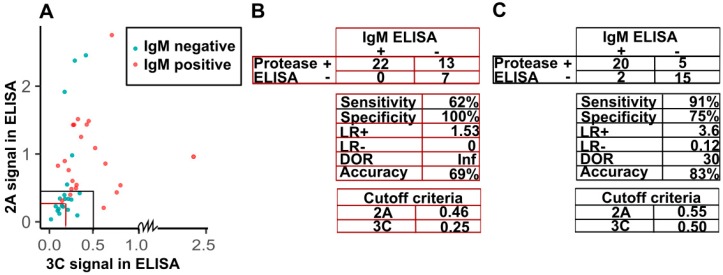
Acute enterovirus infection criteria and test descriptors based on ELISA results. (**A**) Different cutoff criteria based on 2A and 3C signals (based on 6-month children average + 3 × SD (*n* = 20) or the adjusted thresholds) and IgM positivity. In the plot (**A**), IgM positive data points are colored red and control data points are cyan. (**B**) Confusion matrix and test descriptors calculated from the traditional cutoff values based on results from 6-month children comparing the new protease-ELISA test to the IgM ELISA by analysis in R. Sensitivity is the proportion of true positives and specificity is the proportion of true negatives correctly categorized with respect to IgM ELISA by the protease ELISA. LR+ and LR– stand for positive and negative likelihood ratios respectively and DOR the overall diagnostic odds ratio. (**C**) Confusion matrix and test descriptors based on the adjusted diagnosis criteria.

Detection of IgM enterovirus antibodies has been used in the diagnosis of acute or recent enterovirus infections. We decided to study if it is possible to define criteria for detecting acute IgM positive infections in humans based on the detection of IgG class antibodies against enterovirus proteases. As a baseline, we chose to look how the test performed when using criteria based on the 20 presumably naive serum samples from 6-month old children (cutoff set at average signal + 3×standard deviation) (marked with red in Figure 5A). These cutoffs (0.46 and 0.25 for 2A and 3C respectively), while highly specific, gave poor sensitivity (62%) and overall accuracy (69%) due to high number of false positives for the adult data (Figure 5B). Thus, we chose to adjust the criteria based on the IgM ELISA-data. By comparing the 2A and 3C protease antibody reactivities in sera from patients with acute enterovirus infections with sera from healthy controls by IgM-ELISA (Figure 4), we decided upon suitable criteria for the recognition of acute enterovirus infections. We simulated diagnostic cutoff values based on visual inspection for the antibody responses to both proteases and interpreted the data so that if a patient had a 2A signal higher than 0.55 or 3C signal higher than 0.5, the patient was considered acutely infected. Visualization of the dataset is shown in Figure 4 A along with the test descriptors (Figure 5B,C).

Based on the adjusted cutoffs, the protease-ELISA results are highly comparable to the IgM-ELISA results with high accuracy (83%), specificity (75%), and sensitivity (91%) between the two methods. As such, the protease antibody ELISA assays provide a valid and easy method to detect acute enterovirus infections in patient sera.

## 4. Discussion

While antibody responses against enterovirus capsid proteins have been studied widely and used in research and diagnostics, the responses that target viral non-structural proteins, and specifically the proteases, are poorly characterized in humans. The present study shows, for the first time, that both mice and humans produce robust antibody responses against enterovirus proteases 2A and 3C, and that these responses are associated with acute or recent enterovirus infections. This suggests that detection of antibodies against enterovirus proteases could be used as a biomarker of recent infection.

So far, rapid serological diagnosis of acute or recent enterovirus infection has relied on the detection of IgM class antibodies in a single sample. However, the performance of IgM assays can vary considerably depending on several factors. The most important limitation is the occurrence of false negative IgM results due to the lack of clear IgM response in some enterovirus infections [24] or the high number of serotypes (>100) which are difficult to completely cover in a single assay [23]. For example, the IgM assay in this study detected VP1 proteins from EV-A and EV-B enteroviruses and we found that there is a fraction of patients that are IgM negative but have high responses towards the 2A protease. This indicates that those patients may have been missed in the IgM assay due to lack in the sensitivity of the assay. Thus, there is a clear need for improved rapid serological assays to detect acute and recent infections. The results of the present study suggest that detection of IgG class antibodies against viral proteases offers a feasible way to develop new serological assays for this purpose. Protease antibodies peaked and levelled off rapidly after acute phase infection in mice and were also at higher levels in IgM positive human specimens. Interestingly, the use of antibodies against either of the two proteases as an indicator of acute infection in these human samples reached 91% sensitivity and 75% specificity when compared to detection of IgM. However, the aim of the present study was not to validate the use of protease antibodies in enterovirus diagnostics and further work is required to develop such test.

To assess the diagnostic value of the proteases as acute infection markers, it would be important to study the antibodies’ cross-reactivities with other enterovirus proteases. Larger sample set from infected humans should be studied and the relationship between IgM and IgG responses towards the proteases should be examined further. Zhang and collaborators [7] mapped the IgG and IgM antibody responses of EV71-infected human sera to the linear epitopes of the EV71 polyprotein. Patients, who did not produce IgG antibodies towards a group of viral antigens, including 3C, had a more severe infection than the other two groups [7]. In the current study we observed that the correlation between 2A and 3C antibody reactivity was not very high (Cor = 0.55; Figure 4B), indicating that during an acute infection some people raise a higher level of antibodies against one virus protease compared to the other. However, due to the limited number of samples and the information available regarding the patients used in this study, it is hard to draw any comprehensive conclusions, and therefore it would be intriguing to compare the proteases and VP1 IgM antibody responses in a larger sample set. If in a similar manner to the case of EV71 [7], there is a correlation between disease outcome and differences in the immune responses to 3C and other antigens such as 2A, testing for individual protease responses could give valuable information about the severity of the infection.

A recent study used VirScan [25] technology to locate the immunodominant epitopes (against which antibodies were found in >50% of seropositive cases) for the antibodies against the 44 most common viruses that 78 newborn children were seropositive for [26]. This is a reasonably accurate representation of the mother’s IgG fraction, as under normal circumstances, IgM antibodies are generally unable to penetrate the placenta. Viruses in that study included the enteroviruses A, B, and C, as well as rhinoviruses A and B. The immunodominant epitopes for these viruses in the IgG class antibodies were mainly directed against the VP4 and VP1 structural proteins, of which the latter contains the enterovirus and rhinovirus group specific (broad-reactive) epitopes [27,28]. Additionally, antibodies against the 3A non-structural protein were observed among the most common antibodies in their study, indicating that the response against 3A was long-lived [26]. In contrast, IgG antibody responses against 2A and 3C, which do not contain the immunodominant epitopes, could provide a better metric for distinguishing acute infection than antibodies against the structural proteins or 3A.

In addition to the routine diagnostic applications, the detection of antibody responses against enterovirus proteases could offer new opportunities for the evaluation of vaccine efficacy in preclinical and clinical studies. This topic is very timely since new kinds of inactivated poliovirus vaccines are being developed for the end-game of poliovirus eradication and vaccines against other enteroviruses are also in clinical development; e.g., enterovirus 71 vaccines have already been licensed in China and a Coxsackie B virus vaccine is approaching clinical trials [29]. These vaccines are based on highly purified viruses that have been inactivated with formalin and do not contain non-structural proteins. Thus, detection of antibody responses against viral proteases would make it possible to distinguish between responses that have been mounted to the vaccine from those mounted during acute enterovirus infections, offering an option to study vaccine-induced protection against natural infections in a similar manner to the livestock industry where the non-structural proteins 2C and 3ABC have been used to distinguish between immune responses to HFMD infections and HFMD vaccines [15].

The present study has certain limitations. The protease antigens used to detect the IgG responses represented only one enterovirus serotype and screening of antibody response towards proteases from other serotypes was not done. Thus, it is possible that responses against these proteins could differ between infections caused by different serotypes. However, since the proteases are more conserved between different enterovirus serotypes [12] than the VP1 proteins, we expect that such cross-reactivity would occur. Moreover, high protease antibody levels were found in IgM positive human subjects, whose acute infections were most likely caused by a wide range of different enteroviruses (samples were collected during 2016–2019). It is possible that this kind of cross-reactivity could help to cover a wide range of different enteroviruses in one assay, and it should be evaluated in further studies.

## 5. Conclusions

Humans and mice raise antibody responses to enteroviral proteases 2A and 3C after enterovirus infection and this response can be used to differentiate between acute on-going infections and those that occurred previously. After an initial CVB infection in mice, the antibody response towards the proteases is short-lived, peaking at around 7dpi and fading off by 21 dpi. For the human samples, based on our limited dataset of IgM positive acute infections and IgM negative healthy controls, the protease antibody IgG response is a good metric for a recent or on-going infection. Therefore, the use of protease antibody responses in diagnosing enterovirus infections shows promise, although a larger set of samples, preferably from different populations, should be screened to verify the results of the current study and to establish criteria for diagnostic assay.

## Supplementary Materials

The following are available online at https://www.mdpi.com/1999-4915/12/1/78/s1, Table S1: Summary of a previously conducted time course study examining 2A, 3C, and VP1 histological staining in the pancreas of C57BL/6J mice infected with CVB3, Table S2: Interassay variabilities of control samples as percentages in protease ELISA for mouse serum samples. Table S3: Interassay variabilities of controls samples as percentages in protease ELISA for human samples (19 plates in total).

## Appendix A

### Appendix A.1. Mouse Infection and Sample Collection

Approximately eight-week-old mice were infected by intraperitoneal 200 µL injection with Coxsackie B 3 viruses (CVB3) or mock-infected with phosphate buffered saline (PBS). Sera from mock (PBS, *n* = 4), or CVB3 strain Nancy (10^5^ PFU, *n* = 7) infected mice were collected at different time points before and after infection. Animals were also infected with CVB3 at a dose of 10^2^ PFU for harvesting organs. Animal health was carefully monitored. The mice that were infected for organ harvesting were sacrificed at 3 dpi, whereas the other animals were monitored until 21 dpi, unless euthanized due to reaching humane end-point criteria.

### Appendix A.2. Histology

Mice pancreata were recovered from animals sacrificed on 3 dpi, fixed for 24 h in 4% formaldehyde and embedded in paraffin. The formalin fixed tissues were cut into 5 μm sections and immunohistochemical staining was performed with antibodies targeting 3C or VP1 as described earlier [21]. In brief, tissue sections were stained with a biotinylated primary antibodies to VP1 (Dako, clone 5-D8/1, biotinylated by Capra Science, Sweden, and here denoted VP1-bio) or an antibody to 3C [21]. Detection of 3C was performed using a biotinylated goat anti-rat IgG antibody (Vector Laboratories, USA). Finally, biotin was detected using the Vectastain Elite ABC HRP kit (Peroxidase, Vector Laboratories, USA). A dilution of 1:100 was used with the biotinylated VP1 antibody to avoid possible cross-reactivity previously reported with the Dako antibody [30]. Control staining was performed by omitting the primary antibodies in order to further ensure specificity.

### Appendix A.3. Optimization of Protease-ELISA

The optimal conditions for coating ELISA-plates were screened for the different antigens with regards to the coating buffer and antigen concentrations. Also, different dilutions of pooled sera from the infected mice and a human immunoglobulin concentrate Nanogam^®^ were tested to establish a proper analysis concentration range for the sera included in the experiments.

A dilution series from 80 µg/mL to 0.625 µg/mL with 1:2 dilution steps was prepared for 2A, 3C, and 10 µg/mL to 0.5 µg/mL for VP1 proteins and inactivated viruses in phosphate buffered saline (PBS) or 50 mM carbonate buffer pH 9.6. Different secondary anti-mouse-IgG antibodies were tested until one that did not directly bind to the enterovirus antigens were found.

Nanogam has approximately 10 times the concentration of IgG in normal serum and we found that a dilution of 1:10,000 was suitable as a positive control. For mice, we used 1:1000 dilution of pooled infected mice end-point serum (collected at humane end-point or at 21 dpi) as a positive control.

The acute human samples (IgM positive) were first assayed with 1:250, 1:1000, and 1:4000 dilutions and compared to a smaller set of six control sera. Based on this data set, the dilution 1:1000 was chosen as the differences between the groups were greatest with that dilution. Later, when more control serum samples and the DIPP-samples were obtained all the human samples were assayed at 1:1000 dilution. This way the majority of human sample data was assayed twice with matching results.

Interassay variability percentages (IAV) were calculated with R using the following formula within test series:

(A1) $$\mathrm{IAV}\;=\;(\frac{s}{µ})\times 100$$

where *s* = standard deviation and *µ* = mean. IAV values were calculated for each antigen positive and negative controls.

The assay was further optimized before running the bulk of human samples. While optimizing the protease ELISA for humans using Nanogam^®^, there was still greater background for the proteases than for the structural proteins. As a result, different primary antibody incubation temperatures and times were tested. 1 h +37 °C, 2 h RT or o/n at +4 °C were tested and we noticed that with RT or higher temperature, there was significantly more background also in the serumless control wells. As a result, we conducted the human tests performing the serum incubation at +4 °C o/n.

### Appendix A.4. Preparation of ELISA Antigens

The VP1 antigen (CVB3, strain Nancy) were prepared as described in [20]. Briefly, attL-flanked His-tagged VP1 protein coding synthetic genes were ordered from Life technologies and Gateway cloned into pBVboostFGII-vector [31,32] for antigen production. After sequence verification, the VP1 containing plasmids were transferred to *E. coli* BL21 DE3 Star cells, the proteins were expressed by inducing the cells with Isopropyl beta-D-1-thiogalactopyranoside and the produced recombinant proteins were purified from clarified bacterial cell lysate using immobilized metal anion chromatography (Ni-NTA IMAC). The purified VP1 proteins were dialyzed into pH 7.5 50 mM Tris, 150 mM NaCl Tris-buffered saline (TBS) and stored at −70 °C.

The production of recombinant proteases 3C and 2A was carried out as in [21] using *E. coli* strain BL21-AI as expression host. Purification was performed with ÄktaP-100 equipment using IMAC Histrap FF crude columns (GE Healthcare Bio-Science AB). The proteases were dialyzed into 25 mM HEPES buffer (140 mM NaCl, 2.5 mM EDTA, 2 mM TCEP, 2% trehalose, pH 8.0), lyophilized and stored at −20 centigrade.

The adenovirus and parainfluenzavirus antigens were obtained from Heikki Hyöty’s laboratory. These included two parainfluenza antigens (type 1 and type 3, Serion Immunologics BA1216VS, and BA1263FEC respectively) and one Adenovirus antigen (type 2 Hexon, Serion Immunologics, BA128VS).

### Appendix A.5. Virus Culturing

#### Appendix A.5.1. Culturing Viruses for Infecting Mice

The Coxsackievirus B3 strain “Nancy” was grown in HeLa cells for 24–48 h and then cells were freeze-thawed three times. The culture media was collected, centrifuged at 4000 rpm for 5 min and the supernatant was removed and stored at −80 °C. Virus titers were determined by a standard plaque assay technique using HeLa cells.

#### Appendix A.5.2. Culturing Viruses for ELISA

CVB3 ATCC strain Nancy was grown in green monkey kidney cells (Vero) as described previously [6,22]. Viruses were recovered from the clarified Vero-cell culture supernatants by 30% sucrose cushion pelleting (175,000 *g*, 16 h at 4 °C) and were further purified by gelatin affinity chromatography and pelleting through discontinuous 30%/50% sucrose cushion (285,000 g, 16 h at 4 °C). Purified viruses were characterized as described previously [22,33] and the heat inactivation of the purified virus was carried out by incubating the virus at +58 °C for 30 min.

### Appendix A.6. Protease ELISA for the Infected Mice

The antibody response towards CVB3 proteases and structural proteins (VP1 and heat inactivated CVB3; Nancy) were analyzed through indirect ELISA with dilutions ranging from 1:250 to 1:256,000. Assay conditions were optimized for the different antigens using pooled serum samples from CVB3 infected mice and by testing different secondary antibodies.

Nunc Maxisorp plate wells were coated with antigens (in a volume of 50 µL/well: A total of 1 µg/well of 2A, or 3C in PBS pH 7 or 250 ng/well of VP1 or heat inactivated CVB3 virus 50 ng/well in 50 mM carbonate buffer pH 9.6) overnight (o/n) at room temperature (RT). Wells were washed and blocked for 30 min in 250 µL/well of 0.1% BSA in PBS at +37 °C. Next, the serum sample dilutions ranging from 1:250 to 1:64,000 were incubated first at +37 °C for 1 h and then overnight at +4 °C. Subsequently the wells were washed and incubated with 1:1000 dilution of goat anti mouse IgG (Histofine) secondary antibody at +37 °C for 1h. After washing the color reaction was developed by adding 100 µL/well of Sigma OPD substrate solution followed by 30 min incubation at 37 °C before the reaction was stopped with 100 µL/well 0.5 M H_2_SO_4_. Absorbances were read at 490 nm with Victor Wallac plate reader (Perkin Elmer, Turku, Finland).

Similar protease antibody signal fold changes were seen in serum sample diluted from 1:250 to 1:16,000 in CVB3 infected mice, and we therefore decided to show results from the 1:1000 serum dilutions. Stronger responses to VP1 and inactivated virus were detected than those for the proteases and as such these fold changes were calculated using the 1:64,000 dilutions.

The mouse samples were assayed twice and on the second assay round, the same range of sample dilutions was assayed with the proteases, but VP1 and whole virus responses were assayed from 1:32,000 to 1:1,000,000. Coating of the proteases was verified using the protease-specific monoclonal antibodies generated earlier [21].

Pooled endpoint sera from CVB1 and CVB3 infected mice at time of euthanasia (upon reaching the humane end point) or at 21 dpi at 1:1000 dilution was used as positive control on each plate and wells without serum were used as negative controls (Table S2 for interassay variabilities).

### Appendix A.7. Protease ELISA for Human Samples

Optimization was performed using Nanogam^®^, a pooled human IgG product used for treating agammaglobulinaemia. As a negative control for initial screening with Nanogam^®^, 20 serum samples from 6-month-old children from the DIPP-study were screened for 2A, 3C, and VP1 antibodies. The idea was that children of this age would not have maternal antibodies left and most likely would still not have developed their own antibodies against enteroviruses. Additionally, sera from 22 patients diagnosed to have an acute enterovirus infection according to IgM assay were assayed against sera from 20 IgM negative healthy individuals with similar age distribution to test if the protease ELISA can recognize acute infection.

Human samples were assayed as duplicates at 1:1000 dilutions. The protocol was the same as for the mouse samples, with the exception of added parainfluenza and adenovirus antigens and the positive control serum being 1:10,000 dilution for Nanogam^®^ and the primary antibody incubation lasting overnight at +4 °C. The two parainfluenzavirus antigens were coated in the same wells with equimolar amounts, 25 ng of each in a total volume of 50 µL/well and the adenovirus antigen was coated with 50 ng in a volume of 50 µL/well. Both were diluted in PBS. Consult Table S3 for interassay variabilities.

### Appendix A.8. Capture IgM-ELISA for Human Samples

Enterovirus IgM antibodies were analyzed by a standard method used in clinical virus diagnostic laboratory in the Turku University Hospital, as described by [23]. Briefly, the polystyrene wells were coated with anti-human IgM antibodies (0.25 μg/well). Serum samples were added as duplicates in a dilution of 1:100. After 90 min incubation the wells were washed three times with PBS and a mixture of ECHO11, CAV16 and CVB3 antigens, representing Enterovirus A and B species, were added. After 1-h incubation, the wells were washed and incubated with a mixture of rabbit antibodies against ECHO11, CVA16, and CVB3 for one hour. Finally, the color reaction was developed with OPD substrate and stopped with 0.5 M H_2_SO_4_. Absorbance values over three times of the absorbance value of a negative control serum were considered positive.
