# Supplementary material for: Antibody Responses against Enterovirus Proteases are Potential Markers for an Acute Infection
Source: Viruses. 2020 Jan 9;12(1):78. doi: 10.3390/v12010078 (PMC7020046; doi:10.3390/v12010078)
Supplement: Supplementary file 1 [file viruses-12-00078-s001.pdf]

Table S1. Summary of a previously conducted time course study examining 2A, 3C and VP1 histological staining in the pancreas of C57BL/6J mice infected with CVB3.

	Table S1a			Immunohistochemistry positivity		
**Mouse ID**	**CVB3 dose (PFU)**	**DPI**	**Confirmed infected**	**VP1**	**3C**	**2A**
758	100	d3	Yes	+	+	+
1180	100	d3	Yes	+	+	+
1082	100	d5	Yes	+	+	+
1084	100	d5	Yes	+	+	+
1252	100	d5	Yes	+	+	+
834	100	d7	Yes	+	+	-
899	100	d7	Yes	-	-	-
1599	100	d7	Yes	+	+	+
1608	1000	d14	Yes	-	-	-
1609	1000	d14	Yes	-	-	-
1610	1000	d14	Yes	-	-	-
891	100	d21	Yes	-	-	-
473	100	d21	Yes	-	-	-
1044	control	n/a	n/a	-	-	-
1057	control	n/a	n/a	-	-	-

DPI = days post infection. + = positive staining, - = no staining. Infection was confirmed by histological analysis of the pancreas or the measurement of replicating virus in the pancreas by standard plaque assay.

Table S2. Interassay variabilities of control samples as percentages in protease ELISA for mouse serum samples.

Mock/pooled infected sera	2A	3C	VP1	CVB3
**neg.**	3.6	3	3.6	1.9
**pos.**	32.3	13.3		
**CVB3**				
**neg.**	3.6	1.9	1.5	2.2
**pos.**	4	14		

Table S3. Interassay variabilities of controls samples as percentages in protease ELISA for human samples (19 plates in total).

	Para	Adeno	2A	3C	VP1	CVB3
**neg.**	7.62	9.42	13.79	17.61	11.07	14.09
**pos.**	8.28	5.08	8.64	10.56	7.49	9.52
